# Prenatal unhealthy diet, insulin‐like growth factor 2 gene (*IGF2*) methylation, and attention deficit hyperactivity disorder symptoms in youth with early‐onset conduct problems

**DOI:** 10.1111/jcpp.12589

**Published:** 2016-08-18

**Authors:** Jolien Rijlaarsdam, Charlotte A.M. Cecil, Esther Walton, Maurissa S.C. Mesirow, Caroline L. Relton, Tom R. Gaunt, Wendy McArdle, Edward D. Barker

**Affiliations:** ^1^ Centre for Child and Family StudiesLeiden UniversityLeidenThe Netherlands; ^2^ Department of Child and Adolescent Psychiatry/PsychologyErasmus MC‐University Medical Center Rotterdam RotterdamThe Netherlands; ^3^ Department of PsychologyInstitute of Psychiatry, Psychology and NeuroscienceKing's College LondonLondonUK; ^4^ Medical Research Council Integrative Epidemiology UnitUniversity of BristolBristolUK; ^5^ School of Social and Community MedicineUniversity of Bristol BristolUK

**Keywords:** DNA methylation, Avon Longitudinal Study of Parents and Children, diet, conduct problems, attention deficit hyperactivity disorder, *IGF2*

## Abstract

**Background:**

Conduct problems (CP) and attention deficit hyperactivity disorder (ADHD) are often comorbid and have each been linked to ‘unhealthy diet’. Early‐life diet also associates with DNA methylation of the insulin‐like growth factor 2 gene (*IGF2*), involved in fetal and neural development. We investigated the degree to which prenatal high‐fat and ‐sugar diet might relate to ADHD symptoms via *IGF2* DNA methylation for early‐onset persistent (EOP) versus low CP youth.

**Methods:**

Participants were 164 youth with EOP (*n *=* *83) versus low (*n *=* *81) CP drawn from the Avon Longitudinal Study of Parents and Children. We assessed if the interrelationships between high‐fat and ‐sugar diet (prenatal, postnatal), *IGF2* methylation (birth and age 7, collected from blood), and ADHD symptoms (age 7–13) differed for EOP versus low CP youth.

**Results:**

Prenatal ‘unhealthy diet’ was positively associated with *IGF2* methylation at birth for both the EOP and low CP youth. For EOP only: (a) higher *IGF2* methylation predicted ADHD symptoms; and (b) prenatal ‘unhealthy diet’ was associated with higher ADHD symptoms indirectly via higher *IGF2* methylation.

**Conclusions:**

Preventing ‘unhealthy diet’ in pregnancy might reduce the risk of ADHD symptoms in EOP youth via lower offspring *IGF2* methylation.

## Abstract 2

Read the Commentary on this article at doi: 10.1111/jcpp.12649

## Introduction

Conduct problems (CP) and attention deficit hyperactivity disorder (ADHD) commonly co‐occur. Importantly, evidence from family (Faraone, 2000), twin (Thapar, Harrington, & McGuffin, 2001), and molecular genetic (Holmes et al., 2002) studies suggest that that this co‐occurrence denotes a more severe, familial, and heritable entity, compared to either CP or ADHD alone. Children with an early‐onset and persistent pattern of CP represent a particular at‐risk group, as they often show the highest rates of ADHD (Barker, Oliver, & Maughan, 2010), as well as the greatest levels of psychosocial risk exposures in pregnancy (e.g. poverty, maternal anxiety) and the early postnatal years (e.g. harsh parenting, family discordance) (Barker & Maughan, 2009).

One prenatal risk that is a correlate of these psychosocial risks, yet has received far less attention, is diet. ‘Unhealthy diet’ (e.g. high fat/sugar) is of particular interest as it has been reported to associate with both CP and ADHD (Howard et al., 2011; Jacka et al., 2013; Liu, Raine, Venables, & Mednick, 2004; Sonuga‐Barke et al., 2013). A potential mechanism that might help explain the link between ‘unhealthy diet’ and CP and ADHD is the epigenetic process of DNA methylation, which is highly responsive to the nutritional environment (Drake et al., 2012), and also associates with CP‐related phenotypes (Cecil et al., 2014) and ADHD (Schuch, Utsumi, Costa, Kulikowski, & Muszkat, 2015; van Mil et al., 2014; Walton et al., 2016).

Diet has also been shown to influence methylation of the insulin‐like growth factor 2 gene (*IGF2*) (Heijmans et al., 2008), an imprinted gene that lies close to the insulin and tyrosine hydroxylase genes in a genomic region related to the metabolic regulation of glucose homeostasis, cardiovascular functions, and lipid metabolism (Faienza et al., 2010; Ukkola, Sun, & Bouchard, 2001). *IGF2* may be of interest to ADHD as it is a major modulator of placental and fetal growth (Constancia et al., 2002) and also plays an integral role in brain development after birth (Pidsley, Dempster, Troakes, Al‐Sarraj, & Mill, 2012). Animal and human studies report that *IGF2* is associated with developmental abnormalities in the structure and/or function of the cerebellum (Pidsley et al., 2012) and the hippocampus (Chen et al., 2011; Ouchi et al., 2013), both of which are associated with ADHD (Castellanos et al., 2002; Plessen et al., 2006), as well as other psychiatric disorders such as depression and schizophrenia (Yu, Shen, Zeng, Ma, & Hu, 2013).

Periconceptional risk exposure is associated with abnormal brain development (Jensen et al., 2015), with relevance to CP and ADHD (Fairchild et al., 2011; Rubia, Smith, Brammer, Toone, & Taylor, 2005). Moreover, diet‐induced *IGF2* DNA methylation modifications occur specifically during the periconceptional period and may persist well in adulthood (Heijmans et al., 2008). Together, these findings suggest that the long‐term impact of early‐life dietary factors on CP and ADHD may, at least in part, be explained by *IGF2* DNA methylation. The current study simultaneously examined, for early‐onset persistent (EOP) versus low CP youth, the extent to which unhealthy prenatal and postnatal diet (high fat, high sugar) is associated with ADHD symptoms via DNA methylation of *IGF2* (birth and age 7, collected from blood).

## Methods

### Participants

The Avon Longitudinal Study of Parents and Children (ALSPAC) is a prospective study of children born to 14,541 pregnant women residing in Avon, United Kingdom, with an expected delivery date between April 1, 1991 and December 31, 1992 (85% of eligible population (Fraser et al., 2013)). When compared with 1991 national census data, the ALSPAC sample was found to be similar to the UK population as a whole (Boyd et al., 2013). Ethics approval for the study was obtained from the ALSPAC Law and Ethics Committee as well as Local Research Committees. All participants provided informed consent. The study website contains details of all the data that are available through a fully searchable data dictionary: http://www.bris.ac.uk/alspac/researchers/data-access/data-dictionary/.

This study uses a subsample (*n *=* *346, 50% male) from a larger study of DNA methylation in ALSPAC, the Accessible Resource for Integrated Epigenomics Studies (ARIES (Relton et al., 2015), www.ariesepigenomics.org.uk), which follows previously established CP trajectories and has epigenetic data at birth and/or age 7. The CP trajectories, including (a) low (26.9%), (b) childhood‐limited (25.4%), (c) adolescent‐onset (19.7%), and (d) early‐onset persistent (28.0%), have been previously identified and validated (Barker & Maughan, 2009). Specifically, general mixture modeling was used based on the ‘Conduct Problem’ subscale (4–13 years) of the Strengths and Difficulties Questionnaire (SDQ; (Goodman, 2001). This ‘Epigenetic Pathways to Conduct Problems Study’ subsample is comparable to the full trajectory sample (*n *=* *7,218) in terms of environmental risk and psychiatric comorbidity (Barker et al., 2010). DNA methylation data were available for 321 youth at birth and 326 at age 7.

In light of the objective of this study, and to ensure a feasible set of statistical analyses, we included youth in the low and early‐onset CP trajectories, who had complete data for prenatal ‘unhealthy diet’, *IGF2* DNA methylation at birth and ADHD symptoms (*n*_total_ = 164; *n*_low_ = 81; *n*_EOP_ = 83). We did not assess the childhood‐limited or adolescent‐onset as these youth have different developmental risk pathways than the early‐onset and low CP youth (see Barker & Maughan, 2009; Moffitt et al., 2008).

### Measures

#### DNA methylation data

Five hundred nanograms of genomic DNA from cord blood (birth) or peripheral blood (age 7) was bisulfite‐converted using the EZ‐DNA methylation kit (Zymo Research, Orange, CA). The protocol followed the manufacturer's instructions using the recommended alternative incubation conditions for use with Illumina Infinium arrays. Illumina HumanMethylation450 BeadChips (Illumina, San Diego, CA) were run following the manufacturer's protocol with no modifications, and arrays were scanned using an Illumina iScan (software version 3.3.28). Initial quality control of data generated was conducted using GenomeStudio (Illumina; version 2011.1) to determine the status of staining, extension, hybridization, target removal, bisulfite conversion, specificity, nonpolymorphic, and negative controls. DNA methylation data were only available for samples that passed this stage. Samples were quantile normalized using the dasen function within the wateRmelon package (wateRmelon_1.0.3) (Pidsley et al., 2013) in R and batch‐corrected using the ComBat package (Johnson, Li, & Rabinovic, 2007).

We extracted 139 probes that are mapped to *IGF2* or overlapping regions adjacent to *IGF2*, including *INS‐IGF2* (i.e. position 2150687 to 2183864). For each probe, methylation levels were indexed by beta values (i.e. the ratio of methylated signal relative to the sum of the methylated and unmethylated signals). Factor analysis was used in the total sample to establish the covariance structure among the 139 *IGF2* probes in order to extract a smaller set of underlying factors, removing CpGs with low correlations as needed. A three‐factor solution showed the best fit to the data. Full details of the factor analysis procedure and results are provided as online supporting information (Appendix S1 and Table S1). We present findings relating specifically to factor 1 (37 probes) because factor 2 (11 probes) and 3 (5 probes) did not correlate with ‘unhealthy diet’ and ADHD symptoms for the EOP and low CP trajectory. See Appendix S2 for the location of the *IGF2* methylation probes included in this study, and how these are grouped into factors. See Appendix S3 for the correlations between the *IGF2* methylation probes.

#### High‐fat and ‐sugar diet

The Food Frequency Questionnaire (FFQ; (Micali, Northstone, Emmett, Naumann, & Treasure, 2012) was used to assess (a) maternal dietary patterns at 32 weeks of gestation, and (b) what the mother reported feeding to the child at 3, 4.5, and 7 years of age. The FFQ contains a set of questions about the frequency of consumption of a wide variety of food and drink, with higher scores indicating higher frequency of intake. Possible responses were: never or rarely; once in 2 weeks, 1–3 times per week; 4–7 times per week; and more than once daily. Prenatal and postnatal high‐fat and ‐sugar diet scores, indicated by processed food (i.e. fried food, meat pies or pasties, chips) and confectionery (i.e. crisps, chocolate bars, cakes or buns, biscuits) had been previously created using latent factors (Barker, Kirkham, Ng, & Jensen, 2013). For the current analyses, we combined the postnatal diet scores across time (age 3–7) by the use of a latent factor (range factor loadings = .74–.84).

#### Attention deficit hyperactivity disorder symptoms

Attention deficit hyperactivity disorder (ADHD) symptoms were repeatedly assessed (at age 7, 10, and 13) with the Development and Well‐being Assessment (DAWBA; Goodman, Ford, Richards, Gatward, & Meltzer, 2000), a validated semistructured interview. Parents completed open and closed questions about a range of symptoms relevant to youth psychiatric disorders, including ADHD, oppositional defiant disorder (ODD), conduct disorder (CD), generalized anxiety disorder (GAD), and major depressive disorder (MDD). For each disorder, an ordered categorical measure was generated using computer algorithms (Goodman, Heiervang, Collishaw, & Goodman, 2011), comprising six categories indicating the likelihood of each youth having the disorder from level 0 up to level 5. For the current analyses, we created factor scores across time (age 7–13) for ADHD (range factor loadings = .73–.81), ODD (range factor loadings = .58–.82), GAD (range factor loadings = .58–.59), and MDD (range factor loadings = .37–.72).

#### Control variables

We included two types of control variables; repeated measures of cumulative risk and cell type distribution. First, cumulative risk variables were summated into indices spanning two developmental periods (pregnancy and early‐childhood [birth–age 7]) and regressed on all endogenous study variables. For each developmental period, a cumulative risk index had been previously created using latent factor analyses (Cecil et al., 2014), based on maternal reports, covering five risk domains: (a) life events (e.g. death in family, accident, illness), (b) contextual risks (e.g. poor housing conditions, financial problems), (c) parental risks (e.g. parental psychopathology, criminal involvement and substance use), (d) interpersonal risks (e.g. intimate partner violence, family conflict), and (e) direct victimization (e.g. child bullied by peers or physically hurt; available postnatally). We also assessed maternal smoking during pregnancy, which was measured during the first trimester of pregnancy via maternal ratings, using a yes (*n* = 29)/no (*n* = 135) binary variable. However, this variable did not correlate with *IGF2* DNA methylation (see Table 1) and, hence, was not added as a covariate.

**Table 1:** Correlations and descriptive statistics of the variables by low conduct problem youth (above the diagonal, *n *=* *81) and early‐onset persistent conduct problem youth (below the diagonal, *n *=* *83)

	1.	2.	3.	4.	5.	6.	7.	8.	9.	10.	11.
1. Unhealthy diet prenatal	–	.55*	.16	−.02	.03	−.08	.01	.01	.20^1^	.12	.17
2. Unhealthy diet age 3–7 years	.54*	–	.20	−.02	−.01	−.08	−.07	−.02	.13	.15	.15
3. Factor 1 *IGF2* methylation at birth (mean)	.20^1^	.04	–	−.19	−.09	−.05	−.13	.01	.04	.03	.11
4. Factor 1 *IGF2* methylation age 7 (mean)	.03	−.06	.14	–	−.13	−.13	−.09	−.07	−.06	−.11	−.03
5. ADHD age 7–13 years	.18^1^	.07	.27*	.14	–	.35*	.21^1^	.37*	−.06	.10	.18
6. ODD age 7–13 years	.13	.13	.21^1^	.15	.60*	–	.16	.31*	−.12	−.05	.12
7. MDD age 7–13 years	−.07	−.08	.09	−.16	.30*	.40*	–	.40*	−.004	.14	.22*
8. GAD age 7–13 years	−.07	−.15	−.04	−.07	.22^1^	.23*	.45*	–	−.26*	−.14	.10
9. Cumulative risk prenatal	.11	.08	−.01	−.21	−.03	−.04	.10	.07	–	.55*	.12
10. Cumulative risk birth age 7	−.11	.10	−.03	−.30*	−.05	−.02	.28*	.15	.61*	–	.28*
11. Prenatal smoking (yes = 1, no = 0)	.16	.20^1^	.16	−.06	−.14	−.01	.04	−.02	.17	.22*	–
Low CP youth											
Median (interquartile range)	−0.04 (0.91)	−0.11 (0.85)	0.16 (0.02)	0.17 (0.02)	−0.75^a^ (0.56)	−.63 ^a^ (1.03)	−.39 ^a^ (0.95)	−.22 ^a^ (1.16)	−0.17 (0.50)	−2.34^a^ (5.86)	11.1%^b^
EOP CP youth											
Median (interquartile range)	0.09 (0.99)	0.06 (1.45)	0.16 (0.02)	0.17 (0.02)	0.32 (1.20)	.44 (1.23)	−.09 (0.95)	.31 (0.73)	−0.11 (0.57)	0.32 (7.66)	24.1%^b^

EOP, early‐onset persistent; CP, conduct problems.

^1^ *p*‐value = <.10; *p‐value < .05.

^a^Scores were significantly higher for EOP versus low CP youth (*p *<* *.05). *p*‐values are derived from Mann–Whitney–Wilcoxon tests.

^b^Measured as frequency (%) of prenatal smoking for EOP (*n* = 20) versus low CP (*n* = 9) youth (χ^2^ [1] = 3.37, *p* = .067).

Second, we controlled for cell type heterogeneity to estimate cell proportions using DNA methylation data (Houseman et al., 2012). Specifically, *IGF2* DNA methylation scores were residualized for estimated proportions of cells in whole blood (proportion of CD8+ T cells, CD4+ T cells, natural killer [NK] cells, B cells, and monocytes). Granulocytes were removed because the cell type proportions add up to approximately 100%.

### Data analysis

The analysis proceeded in three main steps. In the first step, we tested for developmental interrelationships between ‘unhealthy nutrition’ and ADHD using a multiple group autoregressive cross‐lagged (ARCL) model. We did so by testing the degree to which, for EOP versus low CP, the relationship between high‐fat and ‐sugar diet and *IGF2* DNA methylation differed. CP trajectory differences and sex differences were tested in nested model comparisons using chi‐square difference tests. Two models were estimated in step 1. The first was an unadjusted model, where we did not control for cumulative risks, and the second was an adjusted model, where cumulative risks were regressed on endogenous study variables. In the second step, we tested, for EOP versus low CP youth, the degree to which prenatal high‐fat and ‐sugar diet might indirectly relate to higher levels of ADHD symptoms via *IGF2* DNA methylation at birth. This indirect pathway was programmed in a model constraint statement. Difference between the EOP and low CP were tested by a bootstrapped (see below) difference between the respective indirect pathways (i.e. EOP – Low CP). In the third step, we examined the extent to which high‐fat and ‐sugar diet and *IGF2* DNA methylation are specific risk factors to the development of ADHD symptoms as opposed to other externalizing (i.e. ODD) or internalizing disorders (i.e. GAD, MDD). Cell type was controlled in all models across steps 1, 2, and 3.

Analyses were performed in Mplus version 7.11 (Muthén & Muthén, 1998) using maximum likelihood estimation. Given the small sample size, we used bootstrapped with bias‐corrected 95% confidence intervals (10,000 bootstraps) to derive variance from the empirical distribution of the observed data.

Model fit was first established using the chi‐square statistic. Missing data were handled through full information maximum likelihood. Youth with scores > 3.29 standard deviation from the mean on any study variable were treated as outliers (*n *=* *3) and their scores winsorized (i.e. transformed to match next highest or lowest value).

## Results

### Descriptive statistics

Table 1 contains the correlations and descriptive statistics of the study variables. These statistics are presented separately for the two CP trajectories. Five results are highlighted. First, in line with previous research, EOP children showed higher levels of ADHD symptoms compared to low CP children. However, means and variances for ADHD differed from zero for the two groups (EOP and low CP youth; all *p*‐values < .001). Second, we found that in EOP but not in low CP youth (a) factor 1 *IGF2* mean DNA methylation at birth was positively correlated with ADHD symptoms and (b) factor 1 *IGF2* DNA methylation at age 7 was negatively correlated with postnatal cumulative risk. Third, for the EOP youth, ‘unhealthy diet’ correlated at a trend level with *IGF2* DNA methylation at birth (*r*[83] = .20, *p *=* *.06) and ADHD (*r*[83] = .18, *p *=* *.10). Fourth, for EOP and low CP youth alike, prenatal cumulative risk was highly correlated with postnatal cumulative risk, but for EOP only, higher postnatal cumulative risk was significantly associated with lower *IGF2* DNA methylation at age 7. Fifth, early‐onset CP youth significantly differed from the low CP in ODD, GAD, and MDD (*p *<* *.05); for EOP and low CP youth alike, prenatal and postnatal *IFG2* DNA methylation was not correlated with ODD, GAD, or MDD.

### Step 1: Autoregressive cross‐lagged (ARCL) model predicting ADHD symptoms

**Figure 1:**
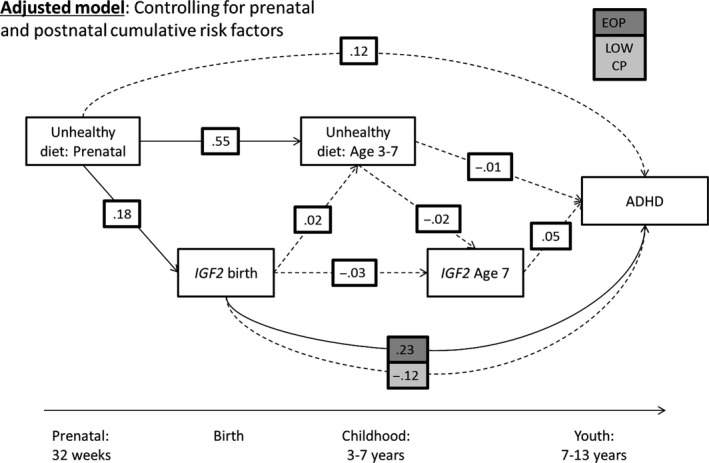
Prospective interrelationships between unhealthy diet, *IGF2* methylation and ADHD for youth with early‐onset persistent (*n *=* *83) versus low (*n *=* *81) conduct problems. Multiple group path analysis. Solid arrowed lines indicate standardized path coefficients that survived bootstrap‐corrected confidence intervals (i.e. significant paths) for EOP versus low CP youth or averaged across all youth. ADHD, attention deficit hyperactivity disorder; EOP, early‐onset persistent; CP, conduct problems.

The unadjusted and adjusted models did not differ in terms of significant path coefficients or model comparisons. Therefore, we present the adjusted model only (see Figure S1 for the unadjusted model). Figure 1 depicts the adjusted ARCL model for ‘unhealthy diet’, *IGF2* DNA methylation, and youth ADHD symptoms. We tested a series of nested model comparisons, where we assessed differences between EOP and low CP youth in: (a) the auto‐regressions; (b) the cross‐lagged associations; (c) the prenatal predictions to ADHD symptoms; and (d) the postnatal predictions to ADHD symptoms of ‘unhealthy diet’ and *IGF2* DNA methylation. The freely estimated model, which served as the comparison model for all nested tests presented below, showed acceptable fit to the data (χ^2^[4] = 6.24, *p *=* *.18). Because sex differences were not identified across the parameters in EOP versus low CP youth ($\Delta \chi_{\mathrm{EOP}}^{2}$[9] = 4.38, *p *=* *.88; $\Delta \chi_{\mathrm{low}}^{2}$[9] = 9.04, *p *=* *.43), we report the results for males and females together.

#### Auto‐regressions

The omnibus auto‐regressions nested chi‐square test constrained two parameters (i.e. stability in diet and *IGF2* DNA methylation, respectively). We found temporal stability in diet but not in *IGF2* DNA methylation, and these estimates did not significantly vary between EOP and low CP youth (∆χ^2^[2] = 5.96, *p *=* *.05), indicating a similar pattern of auto‐regressions across trajectories. This result is statistically significant.

#### Cross‐lagged associations

The omnibus nested chi‐square test for the cross‐lagged associations constrained the parameters of diet influencing *IGF2* DNA methylation and *IGF2* DNA methylation influencing diet. Prenatal diet was associated with *IGF2* DNA methylation at birth for both EOP and low CP youth, and the strength of this association did not significantly differ between the trajectories (∆χ^2^[3] = 1.98, *p *=* *.58).

#### Prenatal predictions to ADHD symptoms

The omnibus nested model chi‐square difference test for the prenatal predictions to ADHD symptoms constrained two parameters (i.e. predictions from prenatal diet and *IGF2* DNA methylation at birth to ADHD symptoms) and these varied significantly between EOP and low CP youth (∆χ^2^[2] = 6.49, *p *=* *.04). Follow‐up difference tests showed an interaction, where the association between *IGF2* DNA methylation at birth and ADHD symptoms at age 7–13 years was significantly higher for EOP versus low CP youth (∆χ^2^[1] = 5.58, *p *=* *.02) (see Figure 1). This is noteworthy given that (a) we had previously shown no DNA methylation difference between the EOP and low CP youth (see Table 1) and that (b) the association between prenatal ‘unhealthy diet’ and ADHD symptoms did not significantly differ between EOP and low CP youth (∆χ^2^[1] = 0.40, *p *=* *.53) and was not significant when averaged across all youth (see Figure 1).

#### Postnatal predictions to ADHD symptoms

The omnibus nested model chi‐square difference test constrained the parameters of postnatal diet and *IGF2* DNA methylation at age 7 predicting ADHD symptoms. These associations did not significantly vary between EOP and low CP youth (∆χ^2^[2] = 1.70, *p *=* *.43) and were not significant when averaged across all youth.

### Step 2: Indirect pathway

For EOP youth, prenatal ‘unhealthy diet’ was indirectly associated with ADHD symptoms via higher *IGF2* DNA methylation at birth. The bias‐corrected confidence interval (via 10,000 bootstraps) for the indirect pathway of prenatal ‘unhealthy diet’ to ADHD symptoms via *IGF2* DNA methylation at birth did not cross zero (*b *=* *.069; 95% CI .003, .206). For low CP youth, the 95% CI of this indirect pathway via *IGF2* DNA methylation did cross zero (*b *=* *−.015; 95% CI −.086, .019). The indirect pathway was different between the EOP and low CP youth: the 95% CI of the difference of the indirect pathways did not cross zero (*b *=* *−.084; 95% CI −.224, −.005).

### Step 3: Other disorders

In light of the findings above, we repeated the ARCL model to examine the extent to which the association of *IGF2* DNA methylation was specific to ADHD symptoms as opposed to other psychiatric disorders (ODD, GAD, and MDD) for EOP versus low CP youth. For EOP and low CP youth, *IGF2* DNA methylation was unrelated to all other disorders (i.e. associations did not survive bootstrapped confidence intervals).

## Discussion

In the present study, we used a longitudinal design to investigate prospective associations between ‘unhealthy diet,’ *IGF2* DNA methylation, and ADHD symptoms in EOP versus low CP youth. Our results showed that prenatal ‘unhealthy diet’ was positively associated with *IGF2* DNA methylation at birth across both EOP and low CP youth. However, only for EOP youth, (a) higher *IGF2* DNA methylation at birth predicted ADHD symptoms; and (b) prenatal ‘unhealthy diet’ was associated with higher ADHD symptoms indirectly via higher *IGF2* DNA methylation at birth.

The present findings showed a statistical interaction, whereby although DNA methylation levels did not differ between the early‐onset and low CP youth, higher levels of DNA methylation were associated with higher symptoms of ADHD for the early‐onset but not for the low CP youth. What could be the reason for this? One could posit that the reason could lie in symptoms of ADHD – the same association would manifest for low CP youth if their levels of ADHD were the same as those of the early‐onset youth. Another potential explanation could lie in the biological vulnerability of CP with ADHD (Beauchaine, Hinshaw, & Pang, 2010). Indeed, we identified an indirect effect where higher prenatal intake of unhealthy fats/sugars associated with increased ADHD via higher *IGF2* DNA methylation, which may suggest a developmental risk pathway for the early‐onset youth alone. It is important to mention that this biological vulnerability may be tapped by other measures of diet (metabolomics) and biology (HPA axis) that are sensitive to stress (Jones, Park, & Ziegler, 2012; Reynolds, Godfrey, Barker, Osmond, & Phillips, 2007).

In this study, we found that for early‐onset youth, higher prenatal ‘unhealthy diet’ was correlated with higher *IGF2* DNA methylation at birth, but higher postnatal cumulative risk exposure was correlated with lower *IGF2* DNA methylation at age 7. Why might DNA methylation associate with the environment differently at birth versus age 7? Findings may reflect two distinct types of risk exposure (i.e. diet vs. cumulative risk), which may differentially influence *IGF2* function. On one hand, diet (or prenatal nutrition) has been found to directly affect the metabolic functions of the gene (Ukkola et al., 2001), showing – in the present findings – higher offspring methylation with higher maternal caloric intake, and also lower methylation in the case of maternal caloric deprivation (Dutch Hunger Winter; Heijmans et al., 2008). On the other hand, more distal influences, such as the cumulative risks examined here (e.g. poverty, family discord), may affect *IGF2* DNA methylation through stress response pathways, such as increased cortisol activity, which has been found to be associated with lower *IGF2* methylation (Vangeel et al., 2015). Given that maternal (or child) stress can co‐occur with different dietary patterns (Heijmans et al., 2008), the effects of cortisol and inflammation (Thorburn, Macia, & Mackay, 2014) on *IGF2* methylation may be promising avenues for future research.

Several limitations should be considered when interpreting the present results. First, this research is correlational in nature; hence, causality cannot be inferred. However, the present research is based on a longitudinal design, which does allow prospective assessment of prenatal and postnatal effects on DNA methylation and can facilitate the use of methods that can strengthen causal inference (e.g. Mendelian randomization) (Pingault, Cecil, Murray, Munafò, & Viding, 2016; Relton & Smith, 2012). Second, the magnitude of the observed associations was not large, and necessitates replication in larger epidemiological samples. Third, all measures except DNA methylation were based on maternal reports. Hence, the temporal stability of diet may be overestimated. However, it is unlikely that the magnitude of the pathways of interest (i.e. prenatal ‘unhealthy diet’ to *IGF2* DNA methylation; *IGF2* DNA methylation to ADHD symptoms) is artificially inflated by shared method variance. The use of in‐depth interviews for the assessment of ADHD symptoms adds to the robustness of our findings. Fourth, the present study did not identify sex differences in the association between *IFG2* methylation and ADHD. Sex differences are nevertheless a promising avenue for future investigation given that boys are generally higher in externalizing problems (such as CP and ADHD). Fifth, the *IGF2* locus is a complex genomic region that produces multiple transcripts from alternative promoters, serves different biological functions, and is differentially expressed in different tissues and at different developmental periods. This gene may also shift from monoallelic to biallelic *IGF2* promoter methylation during development (Issa, Vertino, Boehm, Newsham, & Baylin, 1996); can show sex differences in monoallelic tissue‐specific expression via parent of origin genetic effects (Pidsley et al., 2012); can show loss of imprinting due to diet (Waterland, Lin, Smith, & Jirtle, 2006); and has important functional genomic relationships (Gonzalez‐Rodriguez et al., 2016). Therefore, it will be important to establish the extent to which the present results can be replicated and extended with the addition of these molecular and epigenetic mediators and moderators. Finally, it is important to note that at the bivariate level, the association between prenatal ‘unhealthy diet’, *IFG2* methylation, and ADHD was not significant (all *p *≤* *.10), but became significant in the overall autoregressive cross‐lag model (i.e. when controlling for all other variables in the model). While such a difference can arise when examining bivariate associations (showing the degree of relationship between variables in a pairwise fashion) versus multivariate associations (i.e. which examine a system of predictions; e.g. Tabachnick & Fidell, 2006), the present results should be considered hypothesis‐generating and are in need of replication.

In summary, this study is the first to examine *IGF2* DNA methylation as a potential intermediary biological mechanism in the association between prenatal diet and ADHD symptoms, for early‐onset conduct youth. That we did not find continuity in *IGF2* DNA methylation between birth and age 7 may support ideas focusing on the prenatal maternal health as an important risk for postnatal disease vulnerability (Barker, 1995). For example, a prenatal maternal high‐fat and ‐sugar diet may alter the DNA methylation status of the *IGF2* gene at birth, which in turn, may increase risk for a range of psychiatric and health disorders. The present study highlights pregnancy as being a promising window of opportunity for reducing the risk of ADHD symptoms associated with the nutritional environment and *IGF2* DNA methylation. This is encouraging, given the potentially modifiable nature of nutritional and epigenetic risk factors.

**Key points**

- This population‐based study used a longitudinal design to investigate, in youth with early‐onset persistent (EOP) versus low conduct problems (CP), the interrelations between unhealthy diet and *IGF2* DNA methylation in the prediction of attention deficit hyperactivity disorder (ADHD) symptoms.
- Prenatal unhealthy diet was positively associated with *IGF2* methylation at birth for both the EOP and low CP youth.
- Only for EOP youth, higher *IGF2* methylation predicted ADHD symptoms.
- Only for EOP youth, prenatal unhealthy diet was associated with higher ADHD symptoms indirectly via higher *IGF2* methylation.

## Supporting information


**Appendix S1.** Factor analysis procedure for reducing IGF2 methylation data and results.
**Appendix S2.** Location of IGF2 methylation probes included in the study.
**Appendix S3.** Intercorrelations between the IGF2 DNA methylation probes at birth.
**Table S1.** Confirmatory Factor Model of IGF2 methylation patterns at birth.
**Figure S1.** Unadjusted model not controlling for prenatal and postnatal cumulative risk factors.
